# The Nicotinic Receptor Polymorphism rs16969968 Is Associated with Airway Remodeling and Inflammatory Dysregulation in COPD Patients

**DOI:** 10.3390/cells11192937

**Published:** 2022-09-20

**Authors:** Lynda Saber Cherif, Zania Diabasana, Jeanne-Marie Perotin, Julien Ancel, Laure M. G. Petit, Maëva A. Devilliers, Arnaud Bonnomet, Nathalie Lalun, Gonzague Delepine, Uwe Maskos, Philippe Gosset, Myriam Polette, Anaëlle Muggeo, Thomas Guillard, Gaëtan Deslée, Valérian Dormoy

**Affiliations:** 1 Inserm P3Cell UMR-S 1250, Université de Reims Champagne-Ardenne, 51092 Reims, France; 2 Département des Maladies Respiratoires, CHU de Reims, 51092 Reims, France; 3 Plateforme d’Imagerie Cellulaire et Tissulaire (PICT), Université de Reims Champagne-Ardenne, 51097 Reims, France; 4 Département de Chirurgie Thoracique, CHU de Reims, 51092 Reims, France; 5 CNRS UMR 3571, Unité de Neurobiologie Intégrative des Systèmes Cholinergiques, Institut Pasteur de Paris, Université de Paris Cité, 75006 Paris, France; 6 CNRS UMR 9017, Inserm U1019, Institut Pasteur de Lille, Université de Lille, CHU de Lille, 59000 Lille, France; 7 Département de Biopathologie, CHU de Reims, 51092 Reims, France; 8 Laboratoire de Bactériologie, Virologie, Hygiène Hospitalière, Parasitologie, Mycologie, CHU de Reims, 51092 Reims, France

**Keywords:** COPD, airways, epithelial remodeling, nicotinic receptors, rs16969968, inflammation

## Abstract

Genome-wide association studies unveiled the associations between the single nucleotide polymorphism rs16969968 of CHRNA5, encoding the nicotinic acetylcholine receptor alpha5 subunit (α5SNP), and nicotine addiction, cancer, and COPD independently. Here, we investigated α5SNP-induced epithelial remodeling and inflammatory response in human COPD airways. We included 26 α5SNP COPD patients and 18 wild-type α5 COPD patients in a multi-modal study. A comparative histologic analysis was performed on formalin-fixed paraffin-embedded lung tissues. Isolated airway epithelial cells from bronchial brushings were cultivated in the air-liquid interface. Broncho-alveolar fluids were collected to detect inflammatory mediators. Ciliogenesis was altered in α5SNP COPD bronchial and bronchiolar epithelia. Goblet cell hyperplasia was exacerbated in α5SNP small airways. The broncho-alveolar fluids of α5SNP COPD patients exhibited an increase in inflammatory mediators. The involvement of the rs16969968 polymorphism in airway epithelial remodeling and related inflammatory response in COPD prompts the development of innovative personalized diagnostic and therapeutic strategies.

## 1. Introduction

Chronic obstructive pulmonary disease (COPD) is among the leading causes of mortality and morbidity in the world [1]. Smoking and, more generally, harmful particle inhalation are the main identified risk factors [2,3]. The remodeling of the airways and an abnormal inflammatory response are the hallmarks of COPD [4,5,6,7]. Despite numerous large genetic studies on COPD whole lungs or biological fluids [8,9,10], only alpha-1-antitrypsin deficiency has been translated into health treatment and care [11,12]. 

Genome-wide association studies (GWAS) linked single nucleotide polymorphisms of nicotinic acetylcholine receptors (nAchRs) to pulmonary diseases [9]. The rs16969968 polymorphism is localized at position Chr15q25.1, and codes for the CHRNA5 subunit with the modification D398N (α5SNP) [13,14,15]. Recent studies have unveiled associations between α5SNP and lung cancer [16,17,18], nicotine addiction [19], and COPD independently [20]. This is particularly important since the rs16969968 polymorphism is estimated to be present in about 60% of the worldwide population [20,21]. 

We previously demonstrated using in vivo, ex vivo, and in vitro approaches the contribution of α5SNP in airway epithelial remodeling and the development of emphysema in murine models, by inducing molecular and cellular changes and promoting the inflammatory response [22,23]. In addition, α5SNP nasal polyps were more inflamed and presented secretory cell hyperplasia compared to α5WT. Here, we aimed to study the role of the rs16969968 polymorphism in bronchial and bronchiolar remodeling and immune response in COPD tissues, isolated airway epithelial cells, and broncho-alveolar lavage fluids (BALF) from COPD patients.

## 2. Materials and Methods

### 2.1. Human Subjects

Patients scheduled for fiberoptic bronchoscopy and/or lung resection for cancer (University Hospital of Reims, France) were recruited prospectively (*n* = 44, Supplementary Materials: Table S1) following standards established and approved by the institutional review board of the University Hospital of Reims, France (IRB Reims-CHU 20110612), and included in the cohort for research and innovation in chronic inflammatory respiratory diseases (RINNOPARI, NCT02924818). The study included patients with COPD who gave their consent. At inclusion, age, sex, smoking history, and pulmonary function test results were recorded. All mild, moderate, severe, and very severe stage COPD patients were recruited for all the analyses, except for the immunohistochemistry and the immunofluorescent stainings, where only mild and moderate COPD patients were used. At inclusion, all patients were stable with no acute exacerbation of COPD for at least 4 weeks. Subjects who had ceased smoking for more than 6 months were considered former smokers.

### 2.2. Bronchoalveolar Lavage Fluids (BALF)

The bronchoalveolar lavage fluids were sampled as previously described [24]. We performed the inflammatory mediators’ analysis on the proximal fraction corresponding to the bronchial compartment (*n* = 7).

### 2.3. Human Primary Airway Epithelial Cell Culture

Human primary airway epithelial cells (hAEC) were obtained from bronchial brushings of COPD patients (*n* = 10) to establish air-liquid interface (ALI) cultures as previously described [22,24]. The cells were recovered by scraping the brushes and dissociation using trypsin-versene. They were counted with ADAM (NanoEnTek) according to NanoEnTek instructions. One hundred and fifty thousand cells were seeded on 12-well plates containing 0.4 µm Transwells (Corning, Fisher Scientific, New York, NY, USA) coated with 0.3 mg/mL collagen type IV from the human placenta (Sigma-Aldrich, Saint-Louis, MO, USA). PneumaCult-EX (PnC-Ex, Stem Cell, Vancouver, BC, Canada) medium was used for initial proliferation in the apical and basal chambers. Upon reaching cell confluency, the apical medium was removed, and the basal medium was replaced by PneumaCult-ALI (PnC-ALI, StemCell, Vancouver, BC, Canada) medium. The culture medium was changed every 2 days and the cells were kept for 15 days in incubators at 37 °C, 5% CO_2_.

### 2.4. Genotyping

Epithelial cell pellets from bronchial brushings and tissue sections (4 sections of 20 µm of thickness each) trimmed from formalin-fixed paraffin-embedded (FFPE) lung tissue blocks were processed for DNA purification using the GenElute™ FFPE RNA/DNA Purification Plus Kit, according to the manufacturer’s instructions [22]. The CHRNA5 gene was amplified with DNA polymerase TaKaRa LA Taq (TAKARA Bio Inc., Shiga, Japan) using the following primers: forward 5′-AGTCATGTAGACAGGTACTTCACTCAG-3′, reverse 5′-TGGAAGAAGATCTGCATTTG-3′. The amplification products were digested with the Taq I enzyme, recognizing the following sequence: 5′-TCGA-3′, only present in the α5WT sequence. The digestion products were then separated by agarose gel electrophoresis and the gels were imaged using a LAS-1000 Imager for analysis (Aïda software, Raytest, Courbevoie, France). Eighteen patients were α5WT (41%); 24 were heterozygous α5SNP (54.5%); and 2 were homozygous α5SNP (4.5%).

### 2.5. Immunohistochemistry and Immunofluorescent Stainings

Immunohistochemistry and immunofluorescent stainings were performed on FFPE lung tissues distant from the tumor (*n* = 24). Three μm sections were processed for hematoxylin and eosin staining and analyzed on a white light Eclipse Ci-L microscope (Nikon, Tokyo, Japan) equipped with a DS-Fi2 camera (20× Ph) to assess epithelium height in bronchi and bronchioles. The FFPE lung tissue section slides were deparaffinized and blocked with 10% BSA in PBS for 30 min at room temperature. The tissue sections were then incubated with the primary antibodies as listed in Supplementary Materials: Table S2 overnight at 4 °C in 3% BSA in PBS. After washing with PBS, a second primary antibody was used for 2 h at room temperature (antibodies are listed in Supplementary Materials: Table S2). The sections were washed with PBS and incubated with the appropriate secondary antibodies in PBS for 30 min at room temperature. The DNA was stained with DAPI for 20 min at room temperature. Images were acquired on a Zeiss AxioImageur (20× Ph) with ZEN software (v2.0.0.0 2012, Zeiss, Marly le Roi, France) and processed with ImageJ (v1.53q, National Institutes of Health, Bethesda, MD, USA) for analysis. Five random fields per section were taken for the bronchial analyses. All the structures were imaged to quantify basal cell expression, ciliated cells (primary and motile cilia), and proliferative and secretory cells (Muc5ac, Muc5b, and uteroglobin) for bronchiolar analyses. For each field, a threshold was established by subtracting the background with a rolling ball radius of 50.0 pixels, setting the minimum at 0. Basal, proliferative, and PCC were expressed as a number relative to the total area. Motile cilia are expressed as a percentage of recovery of the epithelium surface, while secretory cells as a normalized mean grey value between the two groups.

### 2.6. Whole-Mount Immunofluorescent Stainings

Methanol-fixed hAEC from ALI cultures were rehydrated by decreasing methanol concentrations (75%, 50%, and 25% methanol/PBT) before a post-fixation with acetone. The cells were then blocked with 10% BSA in PBT (PBS + 1% DMSO + 0.1% Triton) for 2 h at room temperature and incubated for one night at 4 °C in 3% BSA/PBT with the primary antibody anti-Arl13b (17711-1-ap, ProteinTech, Manchester, UK). The DNA was stained with DAPI for 20 min at room temperature. The clarification of the cells was achieved by a glycerol gradient (25%, 50%, and 75%) before mounting the slides. The images were acquired on a Zeiss AxioImager (20× Ph) with ZEN software (V2.0.0.0, 2012, Zeiss, Marly le Roi, France) and processed with ImageJ (v1.53q, National Institutes of Health, Bethesda, MD, USA). Primary and motile cilia recovery were quantified and related to the total area. The lengths were measured as described previously [24].

### 2.7. May-Grünwald-Giemsa Stainings

May-Grünwald-Giemsa (MGG) staining was performed on FFPE lung tissues (*n* = 11). Slides were deparaffinized and rehydrated by decreasing ethanol concentrations (100%, 95%, and 70%). Then, the slides were placed in Jenner Stain Stock solution at 50% (eosin, methylene blue, 26114-01, CliniSciences, Nanterre, France), followed by Giemsa Stain Stock solution at 6% (eosin, methylene blue, azure B, GGS500, CliniSciences, Nanterre, France). The slides were dehydrated using ethanol gradients (95% and 100%) and xylene solution before mounting. The images were acquired on a slide scanner (VS120, Olympus, Tokyo, Japan) with Olyvia software (Olympus OlyVIA 2.9, Tokyo, Japan). Bronchi were imaged and inflammatory cells, namely the eosinophils, basophils, neutrophils, and lymphocytes, were manually counted using QuPath software, Belfast, UK [25]. The epithelial length was set as a reference.

### 2.8. Microbiological Analysis

Endobronchial samples (bronchial aspiration or BALF, *n* = 15) were collected, and extended microbiological culture was performed, as previously described [26,27]. The samples and their dilutions (1/1.000 for bronchial aspiration) were cultured in Columbia blood agar, chocolate agar, Schaedler agar, and Pseudomonas selective cetrimide agar (Thermo Fisher Scientific, Waltham, MA, USA), at 37 ℃ for 48 h for aerobic and 5% CO_2_ cultures and 5 days for anaerobic cultures. All colonies that appeared to be morphologically distinct were quantified as colony-forming unit (CFU) per mL and identified using MALDI-TOF mass spectrometry (MALDI Biotyper^®^, Bruker Daltonics, Billerica, MA, USA). The α-diversity of the airway microbiota was evaluated with the Shannon index (a marker of intra-individual diversity).

### 2.9. Immunoblot Analysis

Cytokines and chemokines expression in BALF (1 mL, *n* = 3 α5WT, and *n* = 4 α5SNP) were assayed by a proteome profiler array according to the R&D system’s instructions (ARY022B). The final detection was obtained by streptavidin-HRP and chemiluminescence. The membranes were then imaged using a LAS-4000 gel imager (Fujifilm, Tokyo, Japan) for analyses (Supplemental Figure S5). The detected signals were digitally quantified using ImageJ. The values were normalized to the positive and negative controls for each membrane. A cut-off was applied considering an interval of 5% between the mean grey values of the range of positive minus negative controls. All inflammatory mediators whose expression was lower than this value were considered undetected. The α5SNP expression was normalized to α5WT and reported to 1. The abundance represented the quantities of inflammatory mediators’ expression in the BALF of COPD patients, including α5WT and α5SNP. It was defined by standardizing the difference between the positive and negative controls of both α5WT and α5SNP COPD patients. All the inflammatory mediators’ expression values were calibrated on this difference in expression in the two groups and then normalized to their negative control. The results were expressed as mean values of each group and reported in the heatmap according to their abundance in BALF. Very high: comparative detection higher than 50% of positive control; high: between 50 and 25%; medium: between 25 and 10%; low: between 10 and 5%; and very low: less than 5%.

### 2.10. Statistics

The data are expressed as mean values ± SEM, and percentages. Differences between groups were determined using the Student’s *t*-test one-tailed or to the hypothetical value of 1.00, representing the reference for the α5WT subjects. For microbiological analysis, Mann–Whitney and Fisher’s tests were used. A *p*-value < 0.05 was considered significant.

## 3. Results

**Figure 1:**
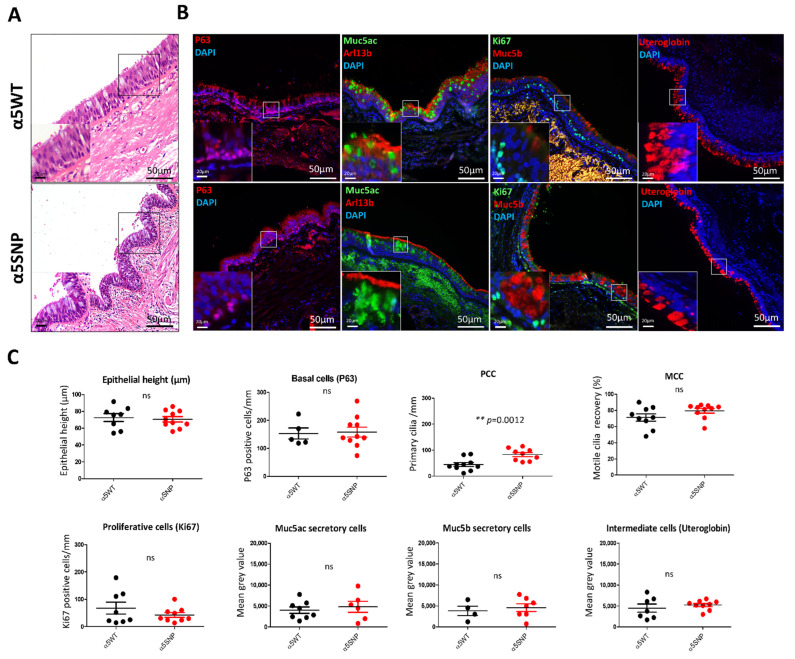
Bronchial epithelial remodeling in rs16969968 (α5SNP) COPD patients. (**A**): Hematoxylin and eosin staining showing the epithelial height of α5SNP and α5WT COPD patients. (**B**): Examples of the microscopic acquisition of immunofluorescent stainings for basal cells (P63, red), ciliated cells (Arl13b, red), proliferative cells (Ki67, green), mucins secretory cells (Muc5ac, green; Muc5b, red), and intermediate cells (Uteroglobin, red). Nuclei are stained in blue (DAPI). Magnification corresponding to the selected area is represented. (**C**): Dot plots (means with SEM) representing measurements of the epithelial height, the number of basal, proliferative, and primary ciliated cells per mm, motile cilia recovery (%), and the mean grey values of mucins (Muc5ac, Muc5b) and uteroglobin-associated fluorescence of α5SNP and α5WT COPD patients. **, *p* <0.01 α5WT vs. α5SNP; ns, non-significant.

We first analyzed the impact of α5SNP on bronchial remodeling, using genotyped lung tissues obtained from COPD patients. The epithelial height and proliferation index did not differ between α5SNP and α5WT respiratory epithelia (Figure 1). 

Interestingly, there was a 54% increase in primary ciliated cells (PCC) (83.30 ± 7.69 PCC/mm of epithelium vs. 44.94 ± 7.66, *p* < 0.01) in α5SNP COPD epithelia (Figure 1b,c and Supplementary Materials: Figure S1). Mucins secretory cells were also increased in α5SNP but did not reach statistical significance (Figure 1b,c). Basal, multiciliated (MCC), and intermediate cells were equally distributed (Figure 1b,c). We further assessed cilia alterations in α5SNP COPD bronchial airway epithelial cells (AEC) isolated from bronchial brushes. There was no difference in the numbers of basal, MCC, and Muc5ac secretory cells between α5SNP and α5WT AEC (Supplementary Materials: Figure S2). Since PCCs are rapidly disassembled in the fresh AEC isolation, we cultured AEC in air-liquid interface (ALI) conditions. We confirmed the alteration of primary ciliogenesis during differentiation with a 67% increase of PCC (75.25 ± 3.23% vs. 50.56 ± 2.61%, *p* < 0.05) in α5SNP COPD patients compared to α5WT (Supplementary Materials: Figure S3). 

**Figure 2:**
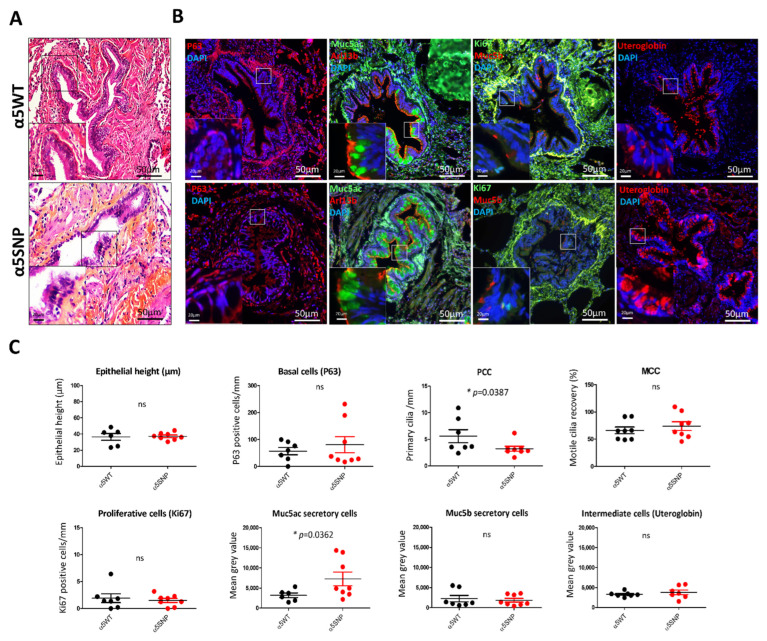
Bronchiolar epithelial remodeling in rs16969968 (α5SNP) COPD patients. (**A**): Hematoxylin and eosin staining showing the epithelial height of α5SNP and α5WT COPD patients. (**B**): Examples of the microscopic acquisition of immunofluorescent stainings for basal cells (P63, red), ciliated cells (Arl13b, red), proliferative cells (Ki67, green), mucins secretory cells (Muc5ac, green; Muc5b, red), and intermediate cells (Uteroglobin, red). Nuclei are stained in blue (DAPI). Magnification corresponding to the selected area is represented. (**C**): Dot plots (means with SEM) representing measurements of the epithelial height, the number of basal, proliferative, and primary ciliated cells per mm, motile cilia recovery (%), and the mean grey values of mucins (Muc5ac, Muc5b) and uteroglobin-associated fluorescence of α5SNP and α5WT COPD patients. *, *p* < 0.05 α5WT vs. α5SNP; ns, non-significant.

Given that COPD is also considered a small airway disease, we assessed bronchiolar remodeling [28]. The epithelial height and proliferation index did not differ between the two groups (Figure 2a,c). 

In contrast, when compared with α5WT, α5SNP bronchiolar epithelium was characterized by a 57% decrease in the number of PCC (3.21 ± 0.46 PCC/mm of epithelium vs. 5.59 ± 1.21, *p* < 0.05) and a 44% increase in Muc5ac secretory cells (7236 mean grey value ± 1717 vs. 3171 ± 583.3, *p* < 0.05) (Figure 2b,c). The basal, MCC, and intermediate cells were not affected (Figure 2b,c). 

We next analyzed the airway microbiota using an extended culture approach and mass spectrometry identification [29]. There was no difference between the α5SNP and α5WT COPD patients’ airway microbiota regarding bacterial distribution and microbiota diversity (Supplementary Materials: Figure S4). 

**Figure 3:**
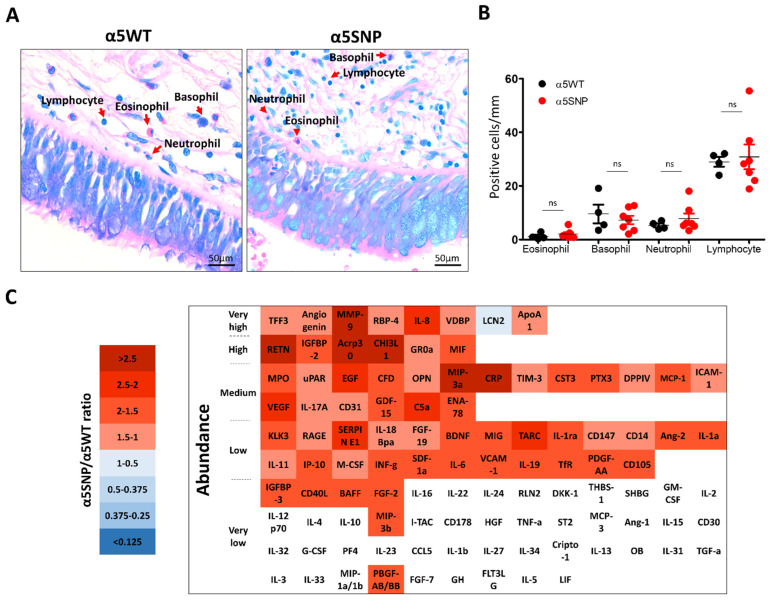
Lung inflammatory response in rs16969968 (α5SNP) COPD patients. (**A**): Microscopic acquisitions showing peribronchial recruitment of immune populations in α5SNP and α5WT COPD patients. (**B**): Dot plot showing the number of eosinophils, basophils, neutrophils, and lymphocytes per mm of epithelium in α5SNP vs. α5WT COPD patients. (**C**): Heatmap presenting the ratios of inflammatory mediators’ expression in broncho-alveolar lavage fluids of α5SNP vs. α5WT COPD patients. Downregulated inflammatory mediators are presented in blue, and upregulated ones are in red. The inflammatory mediators whose expression is lower than the detection cut-off value (5% of positive control) are identified in white. The inflammatory mediators are categorized according to their detected abundance in the broncho-alveolar lavage fluids of COPD patients (from very high, >50% of the detection of the positive control; to very low, <5% of the detection of the positive control). ns, non-significant.

Finally, we analyzed peribronchial recruitment of inflammatory populations (basophils, eosinophils, neutrophils, and lymphocytes), and inflammatory mediators’ (pro- and anti-inflammatory) secretions in BALF obtained from α5SNP and α5WT COPD patients in the proximal compartment (Supplementary Materials: Figure S5). There was no difference in the immune cell distribution (Figure 3a,b). 

TFF3, angiogenin, MMP-9, IL-8, RBP-4, VDBP, Apo-A1, and LCN2 were the most abundant inflammatory mediators detected in COPD patients (Figure 3c). Sixty-two were upregulated in α5SNP COPD BALF (Figure 3c), while total cell counts were similar and below 300,000 cells/mL (data not shown). Six inflammatory mediators (MMP-9, RETN, Acrp30, CHI3L1, MIP-3a, and CRP) were more than 2.5 times upregulated in α5SNP COPD BALF (Figure 3c, and Supplementary Materials: Figure S6).

## 4. Discussion

Taken together, our findings highlight an impairment of PCC and Muc5ac secretory cells in α5SNP COPD patients’ epithelia and dysregulation of inflammatory mediators’ production. We could not evaluate PCC in our previous studies because of the sparsity of basal cells in mice airways. Nonetheless, the epithelial remodeling and the alteration of the inflammatory response were consistent [23]. Further clinical investigations will focus on the quantification of human lung inflammatory populations in α5SNP COPD patients. 

Primary cilia are sensor organelles playing a crucial role in cellular development (proliferation and differentiation) and the reparation process. Anomalies in the structure and/or functions of cilia are responsible for ciliopathies [30]. The number of primary cilia is increased in the bronchial epithelium and decreased in the bronchiolar epithelium of α5SNP COPD patients, suggesting an alteration of the progenitor cell fate toward an arrest of the cell cycle or a loss of stemness. The apparent discrepancy in PCC pointed towards the dual functions of non-differentiated cells in various airway compartments: the alteration of primary cilia may inhibit epithelial repair in bronchi, while it may induce bronchiolar remodeling. These findings are consistent with our previous work regarding the role of primary cilia in COPD pathogenesis and support the implication of α5SNP in airway plasticity in COPD [31,32].

Mucins play an important role as innate immune mediators. They are involved in the clearance of microorganisms and pollutants. The increase of Muc5ac secretion in bronchiolar epithelium without changes in motile cilia recovery suggests an accumulation of mucus in the airway, leading to the airway obstruction described in COPD [33,34,35]. 

Despite no difference in peribronchial recruitment of inflammatory populations between α5SNP and α5WT COPD BALF, six inflammatory mediators were highly upregulated in α5SNP COPD BALF, including MMP-9 and CHI3L1, which are associated with lung remodeling; Acrp30 and CRP, both markers of systemic inflammation; and MIP-3a, which is indirectly related to the activation of NF-kB and STAT3 signaling pathways [36]. Moreover, the large upregulation of detected inflammatory mediators highlights a global dysregulation of the immune response [22,37].

Our analyses were limited by the small sample size for each parameter, although we included a total of 44 patients. There were only two homozygous α5SNP patients (one fiberoptic bronchoscopy and one lung resection), therefore we could not evaluate the additive effect of the polymorphism. We focused on bronchial and bronchiolar remodeling, but it would also be important to investigate alveolar remodeling to complete the analysis. Despite these limitations, we report the first cellular and molecular clues deciphering the genetic impact of α5SNP in COPD patients.

These findings support the involvement of the rs16969968 polymorphism in airway epithelial remodeling and related inflammatory response in COPD patients. The characterization of rs16969968 may contribute to the development of innovative personalized diagnostic and therapeutic strategies in COPD.

## Data Availability

All data generated or analyzed during the current study are available from the corresponding author upon reasonable request.

## Supplementary Materials

The following supporting information can be downloaded at: https://www.mdpi.com/article/10.3390/cells11192937/s1, Figure S1: Identification of primary ciliated cells in bronchial epithelia of α5SNP and α5WT COPD patients; Figure S2: Impact of polymorphism rs16969968 (α5SNP) on cellular distribution in the bronchial epithelium in freshly isolated airway epithelial cells as collected previously [24]; Figure S3: Impact of polymorphism rs16969968 (α5SNP) on cellular differentiation in the bronchial epithelium in human AEC air-liquid interface cultures after 15 days as collected previously [24]; Figure S4: Impact of polymorphism rs16969968 (α5SNP) on bacterial airway microbiota in COPD patients; Figure S5: Inflammatory mediators’ detection in BALF of α5SNP and α5WT COPD patients; Figure S6: Impact of polymorphism rs16969968 (α5SNP) on inflammatory mediators’ detection in BALF of COPD patients; Table S1: characteristics of patients; Table S2: list of antibodies.

## Abbreviations

BALF	Broncho-alveolar lavage fluids
COPD	Chronic obstructive pulmonary disease
FFPE	Formalin-fixed paraffin-embedded
GWAS	Genome-wide association studies
hAEC	human Airway epithelial cell
MGG	May-Grünwald-Giemsa
nAChR	Nicotinic acetylcholine receptor
SNP	Single-nucleotide polymorphism
