# Supplementary material for: The Nicotinic Receptor Polymorphism rs16969968 Is Associated with Airway Remodeling and Inflammatory Dysregulation in COPD Patients
Source: Cells. 2022 Sep 20;11(19):2937. doi: 10.3390/cells11192937 (PMC9563397; doi:10.3390/cells11192937)

Supplementary Materials

The nicotinic receptor polymorphism rs16969968 is associated with airway remodeling and inflammatory dysregulation in COPD patients

Lynda Saber Cherif (1), Zania Diabasana (1), Jeanne-Marie Perotin (1,2), Julien Ancel (1,2), Laure MG Petit (1), Maëva A Devilliers (1), Arnaud Bonnomet (1,3), Nathalie Lalun (1), Gonzague Delepine (1,4), Uwe Maskos (5), Philippe Gosset (6), Myriam Polette (1,7), Anaëlle Muggeo (1,8), Thomas Guillard (1,8), Gaëtan Deslée (1, 2), Valérian Dormoy (1)

Table S1: characteristics of patients. 2

Table S2: list of antibodies. 3

Figure S1: Identification of primary ciliated cells in α5SNP and α5WT COPD patients in bronchial epithelia. 4

Figure S2: Impact of polymorphism *rs16969968* (α5SNP) on cellular distribution in the bronchial epithelium in freshly isolated airway epithelial cells as previously collected [26]. 5

Figure S3: Impact of polymorphism *rs16969968* (α5SNP) on cellular differentiation in the bronchial epithelium in human AEC air-liquid interface cultures after 15 days as previously collected [26]. 6

Figure S4: Impact of polymorphism *rs16969968* (α5SNP) on bacterial airway microbiota in COPD patients. 7

Figure S5: Inflammatory mediators' detection in BALF of α5SNP and α5WT COPD patients. 8

Figure S6: Impact of polymorphism *rs16969968* (α5SNP) on inflammatory mediators' detection in BALF of COPD patients. 9

Table S1: characteristics of patients.

CHRNA5 *rs16969968* (n=44)	α5SNP (n=26)	α5WT (n=18)
**Fiberoptic bronchoscopy, n=20**	n=14	n=6
Age	66.14 ± 9.26	60 ± 9.38
Sex ratio F/M	3F/11M	3F/3M
BMI, Kg/m²	26.94 ± 5.34	24.46 ± 25.92
Smoking: current/former [%]	8 [30.76] /6 [23.07]	3 [50] /3 [50]
Pack-years	38.71 ± 18.99	56.16 ± 25.92
FEV1 (%)	58.57 ± 23.42	55.5 ± 28.80
FEV_1/FVC (%)	52.39 ± 19.22	55.17 ± 21.29
**FFPE lung tissues, n=24**	n=12	n=12
Age	65.75 ± 8.92	69.08 ± 10.31
Sex ratio F/M	3F/9M	3F/9M
BMI, Kg/m²	25.89 ± 4.64	26.5 ± 3.74
Smoking: current/former [%]	5 [41.66] / 7 [58.33]	8 [66.67] /4 [33.33]
Pack-years	49.90 ± 21.76	42 ± 12.42
FEV1 (%)	66.08 ±11.30	74.75 ± 17.70
FEV_1/FVC (%)	64 ± 0.12	59.63 ± 0.06
Tumor types (n=18)	n=9	n=9
Squamous cell carcinoma/Adenocarcinoma/ others [%]	5[55.55]/3[33.33]/1*[11.11]	2[22.22]/7[77.77]/0[0]
Stages: pT1, pT2, pT3/4 [%]	4[44.44]/4[44.44]/1[11.11]	3[33.33]/5[55.55]/1[11.11]

*, large cell lung carcinoma (LCLC)

Table S2: list of antibodies.

Antibodies	Species	Reference	Conditions
P63	Goat	AF1916<br>R&D systems	IHC/IF - 1:100
Arl13b	Rabbit	17711-1-ap<br>ProteinTech	IHC/IF - 1:200
Ki67	Mouse	M7240<br>Agilent Dako	IHC/IF -1:50
Muc5ac	Mouse	NBP2-15196<br>Novus<br>Biological	IHC/IF - 1:100
Muc5b	Rabbit	E-AB-15988<br>Elabscience	IHC/IF - 1:200
Uteroglobin	Rabbit	10490-1-ap<br>ProteinTech	IHC/IF - 1:100

Figure S1: Identification of primary ciliated cells in α5SNP and α5WT COPD patients in bronchial epithelia.

Examples of the microscopic acquisitions of immunofluorescent stainings for ciliated cells (Arl13b, red). Nuclei are stained in blue (DAPI). The arrows show PCC. Magnification corresponding to the selected area is represented.

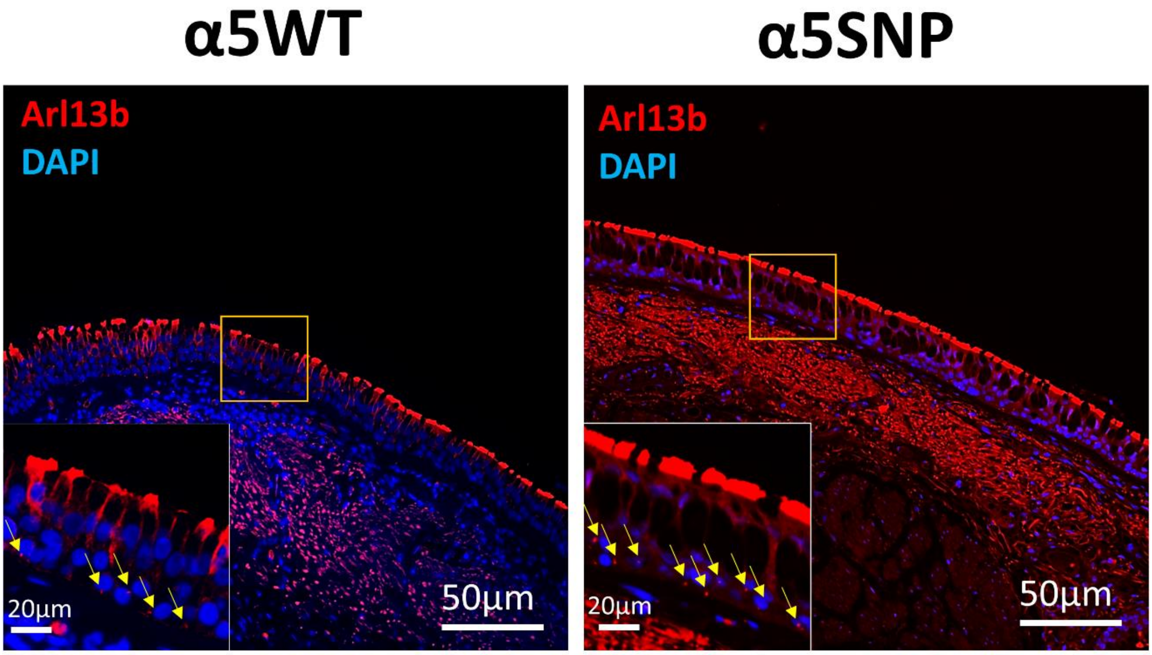

Figure S2: Impact of polymorphism *rs16969968* (α5SNP) on cellular distribution in the bronchial epithelium in freshly isolated airway epithelial cells as previously collected [26]. Dot plots (means with SEM) representing the number of basal cells, MCC, and Muc5ac secretory cells in α5SNP and α5WT COPD patients.

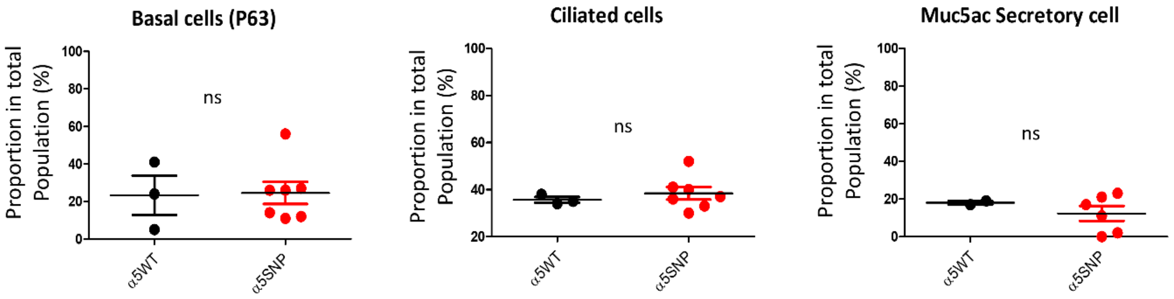

Figure S3: Impact of polymorphism *rs16969968* (α5SNP) on cellular differentiation in the bronchial epithelium in human AEC air-liquid interface cultures after 15 days as previously collected [26]**.**

Dot plots (means with SEM) representing the percentage of PCC, MCC, and cilia lengths in α5SNP and α5WT COPD patients. **, p<0.01 α5WT vs α5SNP; ns, non-significant.

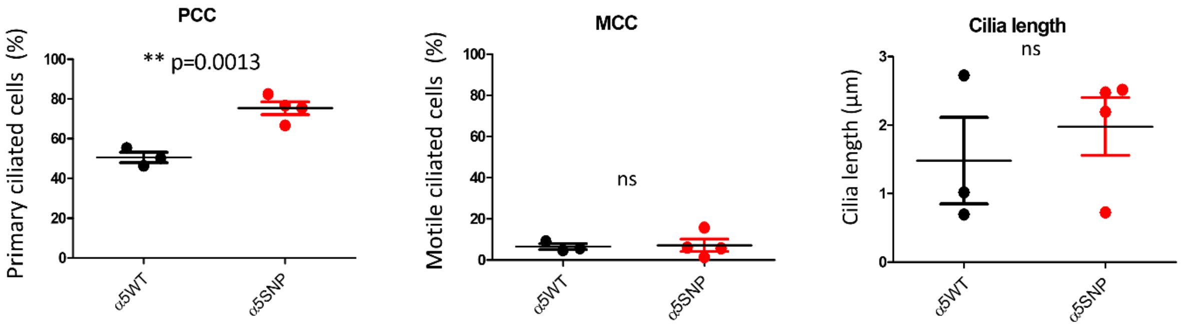

Figure S4: Impact of polymorphism *rs16969968* (α5SNP) on bacterial airway microbiota in COPD patients.

A: Dot plots (means with SEM) representing the number of species per sample in α5SNP and α5WT COPD patients. **B:** Dot plots (means with SEM) representing the alpha diversity of the viable microbiota in α5SNP and α5WT COPD patients. **C:** Dot plots (means with SEM) representing the quantification of CFU (median of CFU per mL) in α5SNP and α5WT COPD patients. **D-E:** Histograms representing phyla and genus distribution in α5SNP and α5WT COPD patients. **F:** Histograms representing the bacteria prevalence (bacteria with less than 10% frequency for the groups are not listed) in α5SNP and α5WT COPD patients.

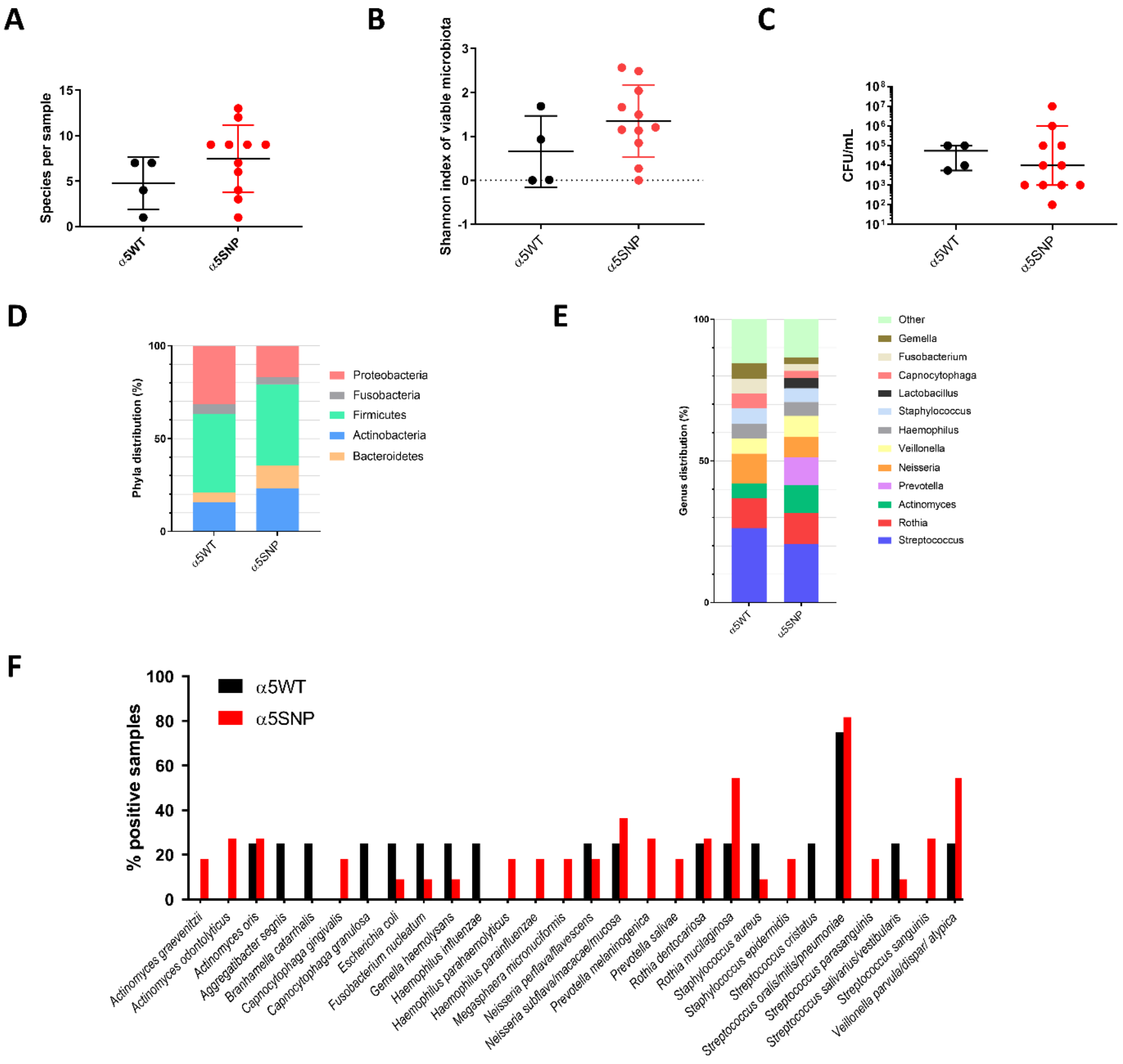

Figure S5: Inflammatory mediators' detection in BALF of α5SNP and α5WT COPD patients.

A: Table listing the position of the inflammatory mediators on the membranes. **B:** Examples of acquisition of nitrocellulose membranes after chemoluminescence detection of BALF in the α5WT and α5SNP COPD.

A

Position	Cytokine	Position	Cytokine	Position	Cytokine	Position	Cytokine	Position	Cytokine	Position	Cytokine	Position	Cytokine
A1, A2	Reference spots	B11, B12	Cripto-1	D1, D2	GR0a	E9, E10	IL-10	F17, F18	IL-34	H1, H2	MPO	I9, I10	TFF3
A3, A4	Acrp30	B13, B14	CST3	D3, D4	GH	E11, E12	IL-11	F19, F20	IP-10	H3, H4	OPN	I11, I12	TfR
A5, A6	ApoA1	B15, B16	Dkk1	D5, D6	HGF	E13, E14	IL-12p70	F21, F22	I-TAC	H5, H6	PDGF-AA	I13, I14	TGFa
A7, A8	Angiogenin	B17, B18	DPPIV	D7, D8	ICAM-1	E15, E16	IL-13	F23, F24	KLK3	H7, H8	PDGF-AB/BB	I15, I16	THBS1
A9, A10	Ang-1	B19, B20	EGF	D9, D10	INF-g	E17, E18	IL-15	G1, G2	OB	H9, H10	PTX3	I17, I18	TNF-a
A11, A12	Ang-2	B21, B22	CD147	D11, D12	IGFBP-2	E19, E20	IL-16	G3, G4	LIF	H11, H12	PF4	I19, I20	uPAR
A13, A14	BAFF	C3, C4	ENA-78	D13, D14	IGFBP-3	E21, E22	IL-17A	G5,G6	LCN2	H13, H14	RAGE	I21, I22	VEGF
A15, A16	BDNF	C5, C6	CD105	D15, D16	IL-1a	E23, E24	IL-18Bpa	G7, G8	MCP-1	H15, H16	RANTES	J1, J2	Reference spots
A17, A18	C5/c5a	C7, C8	CD178	D17, D18	IL-1b	F1, F2	IL-19	G9, G10	MCP-3	H17, H18	RBP-4	J5, J6	VDBP
A19, A20	CD14	C9, C10	FGF-2	D19, D20	IL-1ra	F3, F4	IL-22	G11, G12	M-CSF	H19, H20	RLN2	J7, J8	CD31
A21, A22	CD30	C11, C12	FGF-7	D21, D22	IL-2	F5, F6	IL-23	G13, G14	MIF	H21, H22	RETN	J9, J10	TIM-3
A23, A24	Reference spots	C13, C14	FGF-19	D23, D24	IL-3	F7, F8	IL-24	G15, G16	MIG	H23, H24	SDF-1a	J11, J12	VCAM-1
B3, B4	CD40L	C15, C16	FLT3LG	E1, E2	IL-4	F9, F10	IL-27	G17, G18	MIP-1a/1b	I1, I2	Serpin E1	J23, J24	Negative controls
B5, B6	CHI3L	C17, C18	G-CSF	E3, E4	IL-5	F11, F12	IL-31	G19, G20	MIP-3a	I3, I4	SHBG		
B7, B8	CFD	C19, C20	GDF-15	E5, E6	IL-6	F13, F14	IL-32	G21, G22	MIP-3b	I5, I6	ST2		
B9, B10	CRP	C21, C22	GM-CSF	E7, E8	IL-8	F15, F16	IL-33	G23, G24	MMP-9	I7, I8	TARC		

B

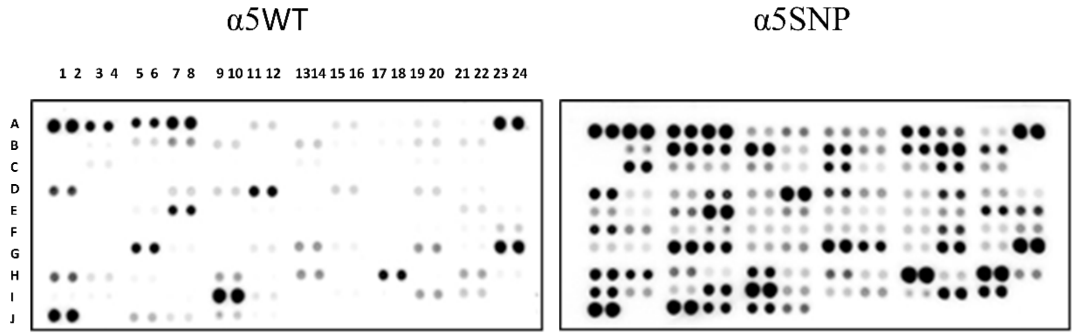

Figure S6: Impact of polymorphism *rs16969968* (α5SNP) on inflammatory mediators' detection in BALF of COPD patients.

Dot plots (means with SEM) showing the mean grey values of the most upregulated inflammatory mediators (Ratio >2.5) in COPD BALF. *, p<0.05 α5WT vs α5SNP; ns, non-significant.

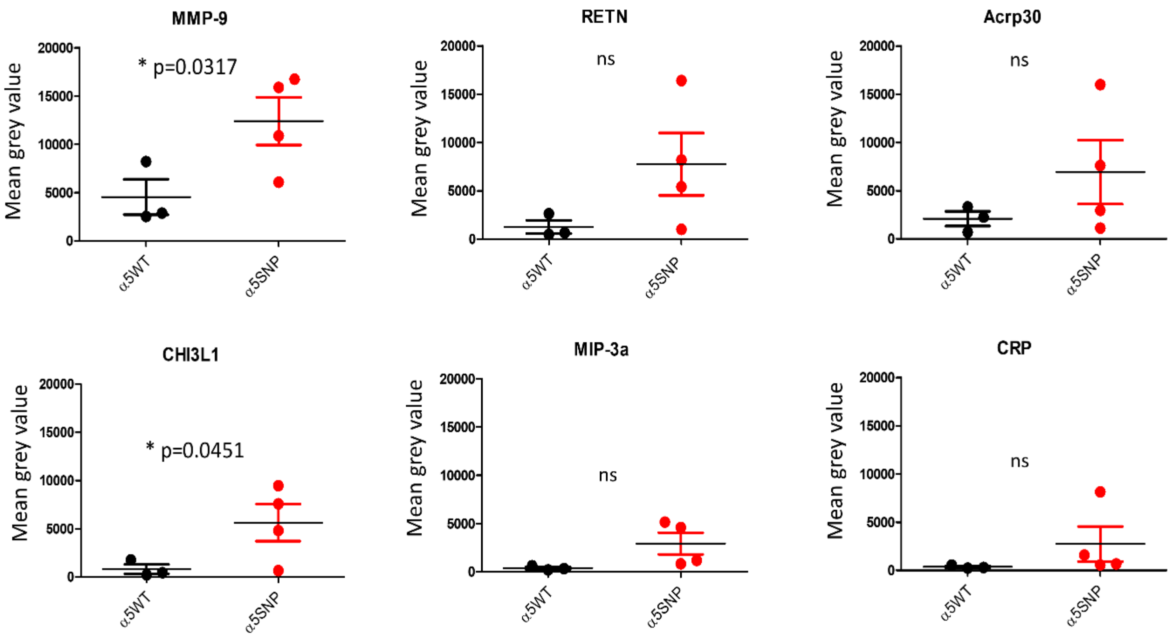

Supplement: Supplementary file 1 [file cells-11-02937-s001.zip › cells-1863001-supplementary.pdf]
